# The relationship between COVID-19-related restrictions and fear of missing out, problematic smartphone use, and mental health in college students: The moderated moderation effect of resilience and social support

**DOI:** 10.3389/fpubh.2022.986498

**Published:** 2022-09-20

**Authors:** Zhun Gong, Yun Lv, Xinian Jiao, Jinhang Liu, Yingjie Sun, Qunzhen Qu

**Affiliations:** ^1^Normal College, Qingdao University, Qingdao, China; ^2^School of Economics and Management, Shanghai Maritime University, Shanghai, China

**Keywords:** COVID-19-related restrictions, fear of missing out, problematic smartphone use, mental health, resilience, social support

## Abstract

As one of the groups most affected by the epidemic, the mental health of college students during the epidemic is a focus of attention in multiple fields. Based on resource conservation theory, this study investigates the impact of COVID-19-related restrictions on college students' problematic smartphone use and mental health from two perspectives, students' individual factors and external environmental factors, and specifically explores the role of fear of missing out (FoMO), resilience and social support in this context. This study used a questionnaire method, and to control for common method bias, a multitemporal data collection strategy was used. The study used online questionnaire distribution, the final sample included 975 Chinese college students (497 males and 478 females), and of these, 10.3% were freshmen, 31.9% were sophomores, 31.6% were juniors, 12.3% were seniors, and 13.9% were postgraduates. The results of this study showed the following: (1) Perceived COVID-19-related strain was positively correlated with perceived FoMO, problematic smartphone use and mental health problems (depression, anxiety, stress) among college students. (2) FoMO partially mediated the relationship between perceived COVID-19-related restrictions and problematic smartphone use, and it fully mediated the relationship between perceived COVID-19-related restrictions and mental health problems. (3) Resilience and social support co-moderated the relationship between FoMO and problematic smartphone use or mental health problems (depression, anxiety, stress).

## Introduction

According to the 49th Statistical Report on the Development of the Internet in China, the number of China's internet users reached 1.032 billion, and the internet penetration rate reached 73.0%. Mobile phones account for 99.7% of internet users and are the main devices used for accessing the internet, and college students are one of the top three main groups, accounting for 17.3% of users. The popularity of the smartphone and the depth of connection users have with it has awakened concerns about its addictive potential (1). Excessive use of smartphones can lead to many undesirable problems, such as addiction-like symptoms and feelings of dependency, which is termed problematic smartphone use (2). Numerous studies have shown a strong link between problematic smartphone use and mental health, especially during the COVID-19 pandemic (3–5). As one of the groups most affected by the epidemic, students' mental health is a major focus of attention in multiple areas because their learning styles and lifestyles have been significantly altered by COVID-19-related restrictions during the COVID-19 pandemic. Therefore, it is necessary to explore the mechanisms underlying the rise in problematic smartphone use and mental health problems among college students during the epidemic and to identify the protective factors that exist between them.

As the COVID-19 pandemic continues to lengthen, the sense of panic caused by the unknown nature of the epidemic is diminishing, and countries are entering the post-epidemic era one after another. As a result, researchers have investigated whether COVID-19-related restrictions, such as home isolation, community closures, online working and public place closures, have had an impact on people's mental health (6). According to self-determination theory, human behavior is divided into self-determined and non-self-determined behavior with three basic psychological needs, autonomy, competence and relationship, and considers drives, intrinsic needs and emotions as sources of motivation for self-determined behavior (7, 8). COVID-19-related restrictions are force majeure measures arising from COVID-19, which are non-self-determined behaviors that undermine the social environment and, to some extent, the three basic needs that could have been met. However, the satisfaction of basic needs is a guarantee of a stable level of mental health. Studies have shown that during the epidemic, there was a significant increase in levels of anxiety and depression among the student population, and the amount of time spent on screens per day changed significantly from the pre-epidemic period (9–12). The reasons for this have been explored by numerous scholars, with students' lack of self-control, the use of online teaching and learning, and the adverse effects of unmet psychological needs and increased isolation of students due to COVID-19-related restrictions all being risk factors for increased problematic smartphone use among students (13). While the current post-epidemic era has seen researchers turn their attention to the range of effects of COVID-19-related restrictions, the underlying mechanisms are still less well-documented, and it remains to be seen how the lack of needs associated with COVID-19-related restrictions has led to more problematic smartphone use and mental problems.

How do COVID-19-related restrictions trigger problematic smartphone use and mental health problems? The problematic smartphone use associated with COVID-19-related restrictions may be different from avoidance-induced mobile phone addiction (13). The increased use of mobile phones during the COVID-19 blockade somewhat alleviated the lack of relational needs, as people used their phones frequently to socialize with friends and family and to obtain epidemic-related information and real-time news from the outside world, but while this alleviated the fear of lack of information, it also created more anxiety, which in turn exacerbated problematic smartphone use (11). Tandon et al. (14) suggests that frequent use of social media to access information about the outside world and others sometimes creates jealousy, and smartphone is one of the most important channels for social media use. That is, the contrast and disparity between the blocked restrictions of one's area and the freedom of others triggers fear of missing out (FoMO) and exacerbates problematic smartphone use. FoMO is a diffuse anxiety caused by an individual's fear of missing out on a novel experience or positive event for others (15). For example, it has been shown that during an epidemic, people are more likely to experience FoMO through online information because of the reduction in offline activities and the abundance of online content due to restrictions (16). Regarding the association between FoMO and problematic smartphone use and mental health problems, according to self-determination theory, individuals experience impaired self-regulation, or FoMO, when their basic psychological needs are not met (15). It has been shown that the lack of relational needs caused by the associated limitations of the epidemic negatively predicts FoMO (6). COVID-19-related restrictions break down people's autonomy, competence and relatedness, and spatial and social restrictions compromise these three basic needs, creating FoMO. In addition, a study by Koban confirmed that an increase in epidemic-related FoMO can affect a person's level of mental health and contribute to daytime fatigue (17). Thus, FoMO may mediate the relationship between COVID-19-related restrictions and problematic smartphone use or levels of mental health.

Although the stress associated with the perceived epidemic and FoMO can increase students' problematic smartphone use and affect their mental health, not all students are severely affected by COVID-19. Conservation of resources theory suggests that individuals have a tendency to strive to acquire, maintain, nurture, and protect the resources they value (18, 19). That is, people use the key resources they have to cope with stressful situations in their current environment while also actively constructing and protecting their existing resource reserves to cope with possible future stressful situations. In a study by Liu and others, resilience and social support were identified as important protective factors for college students in coping with the epidemic after returning to school in the post-epidemic era (20).

Resilience is an individual characteristic that is both variable and stable, reflecting an individual's ability to cope positively with adversity and recover quickly. Resilience has been shown to improve students' environmental adaptability (21). The dynamic model of resilience suggests that resilience is a potential and that students have multiple psychological needs as they grow and that when these psychological needs are met, they develop psychological characteristics that can be transformed into internal resources for the individual (22). COVID-19-related restrictions are the inevitable pressures brought about by changes in the external environment. According to conservation of resources theory (18), people use the key resources they possess to cope with stressful situations in their current environment, and the resilience possessed by college students as they develop during their formative years is a collection of key resources. Resilience is one of the protective resources. Since the beginning of the epidemic, it has been empirically demonstrated that people with high resilience have more stable levels of emotional and mental health during the epidemic and have less problematic smartphone use (23, 24).

Raschke and Helen (25) suggest that social support refers to the care and support people feel from others. Social and emotional support and having good social relationships help to reduce depressive symptoms, control emotions, reduce behavioral problems and increase students' resilience (26). Because of COVID-19-related restrictions for college students, closure is the most immediate measure. According to Bronfenbrenner's ecosystem theory, the university campus is an important microsystem for students at this time. The school, as the main place of activity for college students during closure, and the social support felt on campus are important breakthroughs to compensate for students' psychological needs. In turn, according to the dynamic model of resilience and self-determination theory, social support is an important means of forming and enhancing students' resilience (22). Also, according to conservation of resources theory, social support from school, family, and friends completes the student's psychological protective resources and allows for a constant source of protection against COVID-19 crises, so it may work in tandem with resilience to collectively and directly reduce the impact of FoMO on mental health and problematic smartphone use.

**Figure 1:**
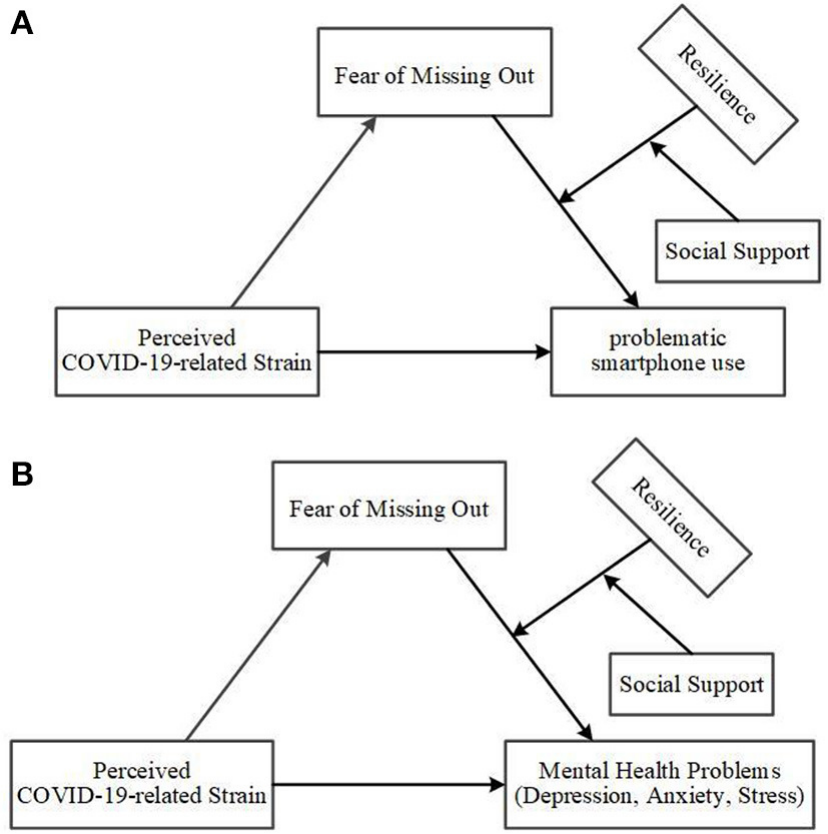
**(A)** The moderated moderated-mediation model of problematic smartphone use. **(B)** The moderated moderated-mediation model of mental health problem.

Building on previous theoretical and research foundations, we attempted to extend previous research by constructing two moderated moderated-mediation models (Figure 1). Problematic smartphone use and mental health problems were two important research variables during the COVID-19, reflecting the negative effects of the COVID-19 on students from both the behavioral (extrinsic) and psychological (intrinsic) sides. The design of the two independent models allows for a more distinct presentation of the behavioral and psychological effects, and because of the dual moderator variable design, this also makes the model more concise and intuitive, better presenting the focus of this study. The model explores the relationship between COVID-19-related restrictions and problematic smartphone use and mental health among college students and its internal mechanisms and explores its moderating role between FoMO and problematic smartphone use or mental health among college students in terms of both the individual's own (resilience) and external forces (social support). This study aims to test the following hypotheses:

H1a: Perceived COVID-19-related restrictions is positively associated with problematic smartphone use among college students.
H1b: Perceived COVID-19-related restrictions is positively associated with mental health problems (depression, anxiety, stress) among college students.
H2: Perceived COVID-19-related restrictions is positively associated with FoMO.
H3a: FoMO mediates the association between perceived COVID-19-related restrictions and problematic smartphone use among college students.
H3b: FoMO mediates the relationship between perceived COVID-19-related restrictions and mental health problems (depression, anxiety, stress) among college students.
H4a: The resilience and social support levels of college students co-moderate the relationship between FoMO and problematic smartphone use; that is, the resilience and social support levels of college students can effectively mitigate the effect of FoMO on problematic smartphone use.
H4b: The level of resilience and social support of college students co-moderates the relationship between FoMO and mental health problems (depression, anxiety, stress); that is, the level of resilience and social support of college students can effectively mitigate the effect of FoMO on mental health problems (depression, anxiety, stress).

## Materials and methods

### Sample

This study used a questionnaire method. To control for common method bias, a multitemporal data collection strategy was used, with data on perceived COVID-19-related restrictions, FoMO, resilience and social support collected at time point T1 (February, 2022) and data on problematic smartphone use and mental health problems collected at time point T2 (April, 2022). To ensure the validity of the measure, Osborne and Costello (27) suggested that the sample size should be 5–10 times the total number of question items. In this study the total number of question items was 97, therefore, we considered it appropriate to recruit about a thousand participants. And the study used online questionnaire distribution. Drawing on Elhai et al. (28), we used the popular Chinese social networking app WeChat to invite participants. We first arranged and recruited the heads of each year at several universities and invited their classmates through the heads. Prior to the survey, all students were informed that the study would be conducted anonymously and that their information would be kept confidential. Interested participants received an online informed consent form. Each participant receives a reward for completion in the amount of RMB 2.

In this study, a series of questionnaires were distributed to 1,003 college students from China. Of the participants, 28 did not complete the surveys and were excluded from the analysis. Thus, the final sample included 975 participants, of which 497 were from males and 478 were from females. In these students, 10.3% were freshmen, 31.9% were sophomores, 31.6% were juniors, 12.3% were seniors, and 13.9% were postgraduates. There were 13.44% participants reporting that they lasted for <2 weeks of duration of closure during the epidemic of COVID-19, 26.46% were “half to one month,” 26.15% were “one to one and a half months,” 10.97% were “one and half to two months,” 22.97% were “two months and over.”

### Measures

#### COVID-19-related restrictions

COVID-19-related restrictions data used the Perceived COVID-19-related Strain Scale developed by Wegmann and contains 12 questions divided into five dimensions: experienced strain due to social contact restrictions, restrictions in the working context, childcare restrictions, travel restrictions and health issues (6). Each of the restrictions and consequences were rated on a 5-point Likert scale (1 = “not at all burdensome” to 5 = “very burdensome”). The scale has good reliability and validity, as verified by the study by Wegmann et al. In the current study, the scale was modified to a more relevant expression for the experiences of college students depending on the research context, for example, “restrictions on campus life, such as the ban on delivery takeaways.” The Cronbach's alpha in this study was 0.893.

#### Fear of missing out

FoMO was determined by one-dimensional scales containing 10 questions scored on a scale of 1–5, with 1 being “Not at all true of me” and 5 being “Extremely true of me” (29). The higher the score is, the higher the level of FoMO. The results of the analysis of data from adults and adolescents in the USA, Spain, China and Turkey showed that the reliability of the scale was good, with internal consistency coefficients above 0.83. The Cronbach's alpha in this study was 0.906.

#### Problematic smartphone use

Problematic smartphone use was measured using the Mobile Phone Addiction Inventory (MPAI), a 17-question scale (30). The scale consists of four dimensions: inability to control craving, anxiety and feeling lost, withdrawal and escape, and productivity loss. A 5-point Likert scale was used, with 1 = “not at all,” 2 = “rarely,” 3 = “occasionally,” 4 = “often,” and 5 = “always.” The higher the scale score is, the higher the index of mobile phone addiction. In the current study, the overall Cronbach's alpha was 0.953, the inability to control craving was 0.902, anxiety and feeling lost was 0.868, withdrawal and escape was 0.774, and productivity loss was 0.840.

#### Resilience

Resilience was assessed using the Resilience Scale CD-RISC, a revision of the Connor-Davidson Toughness Scale (CD-RISC) by Xiao-Nan Yu of the Chinese University of Hong Kong, which consists of 25 items that are assessed on a 5-point Likert scale ranging from 1 to 5 corresponding to “not true at all” to “true nearly all of the time” (31). The scale consists of 3 dimensions: perseverance, self-improvement and optimism. The higher the scale score is, the stronger the resilience. In the current study, the overall Cronbach's alpha was 0.979, perseverance was 0.961, self-improvement was 0.941 and optimism was 0.870.

#### Social support

The Perception Social Support Scale (PSSS) was developed by Blumenthal in 1987 (32). The PSSS has 12 items and 3 subscales, including the dimensions of family support, friend support, and support from others. A seven-point Likert scale was used, from 1 = “extremely disagree” to 7 = “extremely agree.” The higher the score is, the higher the perceived social support. In this study, the overall Cronbach's alpha was 0.977, family support was 0.940, friend support was 0.941 and other support was 0.938.

#### Mental health problems

The Depression Anxiety and Stress Scale (DASS-21), a short version of the DASS, was originally developed by Lovibond et al. in 1995, and the simplified version of the DASS-21, revised in 2010, was used in this study (33). The full scale consists of 21 items scored on a 4-point Likert scale from 0 to 3 corresponding to “not at all” to “always.” The higher the score is, the lower the level of mental health. In this study, the overall Cronbach's alpha was 0.978, stress was 0.930, anxiety was 0.937 and depression was 0.941.

#### Statistical method

Data entry and analysis were conducted using SPSS 26.0 and PROCESS 3.3. First, descriptive statistics, independent samples *t* tests, one-way ANOVA and correlation analysis were conducted on the main variables. Second, we used the PROCESS 3.3(Model 18) in SPSS 26.0 to examine the mediating role of FoMO in the relationship between COVID-19-related restrictions and problematic smartphone use or mental health problem, as well as the moderated moderating role of resilience and social support in the relationship between FoMO and problematic smartphone use or mental health problem.

## Results

### Descriptive values and correlation analysis

To explore differences in student scores on problematic smartphone use and mental health problems by gender, age, grade, and duration of closure, differences tests were conducted (see Table 1). Independent sample *t* tests revealed significant gender differences in the following variables: problematic smartphone use (*t* = 5.80, *p* < 0.001), and mental health problems (*t* = 9.14, *p* < 0.001). Problematic smartphone use, and mental health problems were significantly higher in men than in women. And one-way ANOVA revealed that grade level was significant difference for problematic smartphone use (*F* = 6.69, *p* < 0.001) and mental health problems (*F* = 12.08, *p* < 0.001), and *post hoc* analysis of multiple comparison results showed postgraduates scored significantly lower than other grades on problematic smartphone use and mental health problems. Moreover, the duration of closure was significant difference for problematic smartphone use (*Welch* = 6.84, *p* < 0.001) and mental health problems (*Welch* = 25.85, *p* < 0.001). Of these, *post hoc* analysis of multiple comparison showed that problematic smartphone use was significantly lower for “two months and over” than for “less than one and a half months” closure. “One and a half months and over” scored significantly lower than “less than one and a half months” on mental health problems. However, we also found no significant age differences in problematic smartphone use (*F* = 1.10, *p* > 0.05), and mental health problems (*F* = 0.80, *p* > 0.05).

**Table 1:** Difference test for gender, age, grade, and duration of closure (*M* ±*SD*).

**Variable**	**Group**	**Problematic smartphone use**	**Mental health problem**
Gender	Male	3.63 ± 0.80	2.70 ± 0.81
	Female	3.30 ± 0.92	2.21 ± 0.86
	*t*	5.80^***^	9.14^***^
Age	18–20	3.42 ± 0.80	2.40 ± 0.81
	21–22	3.51 ± 0.90	2.49 ± 0.87
	23–24	3.45 ± 0.83	2.47 ± 0.87
	25 and over	3.32 ± 1.03	2.36 ± 0.98
	*F*	1.10	0.80
Grade	①Freshmen	3.54 ± 0.90	2.50 ± 0.91
	②Sophomores	3.49 ± 0.89	2.53 ± 0.85
	③Juniors	3.59 ± 0.79	2.57 ± 0.83
	④Seniors	3.41 ± 0.93	2.47 ± 0.89
	⑤Postgraduates	3.15 ± 0.89	2.00 ± 0.80
	*F*	6.69^***^	12.09^***^
	*LSD*	⑤<①②③④	⑤<①②③④
Duration of closure	①Less than two weeks	3.52 ± 0.98	2.56 ± 0.93
	②Half to one month	3.63 ± 0.78	2.70 ± 0.78
	④One to one and a half months	3.57 ± 0.79	2.61 ± 0.82
	⑤One and half to two months	3.38 ± 0.99	2.28 ± 0.91
	⑥Two months and over	3.18 ± 0.88	2.04 ± 0.79
	*W*	6.84^***^	25.85^***^
	*Tamhane*	⑤<①②③	⑤④<①②③

*p <0.05, **p <0.01,

*** p <0.001.

Due to heterogeneity of variance, it was reported as Welch values in duration of closure difference tests and as F values for the others.

To explore the correlations between the variables, this study used correlation analysis and the results are shown in Table 2. Perceived COVID-19-related restrictions were positively correlated with fear of missing out (*r* = 0.59, *p* < 0.001), problematic smartphone use (*r* = 0.44, *p* < 0.001) and mental health problems (*r* = 0.27, *p* < 0.001).

**Table 2:** Correlations and means of study variables (*N* = 975).

	***M* ±*SD***	**1**	**2**	**3**	**4**	**5**	**6**
1. COVID-19-related restrictions	3.81 ± 0.74	1					
2. Fear of missing out (FoMO)	3.59 ± 0.83	0.59^***^	1				
3. Resilience	3.49 ± 0.92	−0.06	−0.16^***^	1			
4. Social support	4.84 ± 1.54	−0.07^*^	−0.19^***^	0.49^***^	1		
5. Problematic smartphone use	3.47 ± 0.88	0.44^***^	0.69^***^	−0.19^***^	−0.23^***^	1	
6. Mental health problem	2.46 ± 0.87	0.27^***^	0.52^***^	−0.40^***^	−0.46^***^	0.60^***^	1

* p <0.05, **p <0.01,

*** p <0.001.

### Moderated moderated-mediation analysis

To investigate the mechanisms and boundaries of the effects of perceived COVID-19-related restrictions on problematic smartphone use, this study used SPSS PROCESS 3.3 to construct a moderated mediation model (Model 18), and the results are shown in Table 3. Perceived COVID-19-related restrictions was positively associated with problematic smartphone use among college students (β = 0.45, *p* < 0.001), therefore H1a is accepted. Perceived COVID-19-related restrictions was positively associated with fear of missing out (β = 0.60, *p* < 0.001), and fear of missing out was positively associated with problematic smartphone use (β = 0.58, *p* < 0.001), thus H2 and H3a are accepted. At the meantime, the direct effect of perceived COVID-19-related restrictions on problematic smartphone use was significant under the influence of FoMO (β = 0.09, *p* < 0.01). It indicates that fear of missing out partially mediates the association between perceived COVID-19-related restrictions and problematic smartphone use among college students. Moreover, a 3-way interaction of FoMO, resilience and social support was positively associated with problematic smartphone use (β = 0.10, *p* < 0.05), which means that the resilience and social support levels of college students co-moderate the relationship between fear of missing out and problematic smartphone use, and H4a is accepted.

**Table 3:** The moderated moderated mediating tests of problematic smartphone use.

**Outcome**	**Predictors**	**Fit Indices**		**Coefficients**	
		***R* ^2^**	***F***	**β**	***t***
Problematic smartphone use	COVID-19-related restrictions	0.26	67.90^***^	0.45	16.31^***^
	Gender			−0.14	−5.05^***^
	Age			0.03	0.93
	Grade			−0.08	−2.06^*^
	Duration of Closure			−0.15	−5.25^***^
FoMO	COVID-19-related restrictions	0.38	121.25^***^	0.60	23.61^***^
	Gender			−0.08	−3.15^**^
	Age			0.06	1.83
	Grade			−0.11	−3.31^**^
	Duration of Closure			−0.11	−4.11^***^
Problematic smartphone use	COVID-19-related restrictions	0.51	82.34^***^	0.09	2.96^**^
	FoMO			0.58	17.76^***^
	Resilience			−0.04	−1.18
	Social support			−0.08	−2.31^*^
	FoMO × Resilience			−0.02	0.46
	FoMO × social support			0.02	0.48
	Resilience × social support			−0.12	−3.24^**^
	FoMO × resilience × social support			0.10	2.17^*^
	Gender			−0.08	−3.43^***^
	Age			−0.01	−0.36
	Grade			−0.004	−0.14
	Duration of closure			−0.08	−3.35^***^

* p <0.05,

** p <0.01,

*** p <0.001.

**Figure 2:**
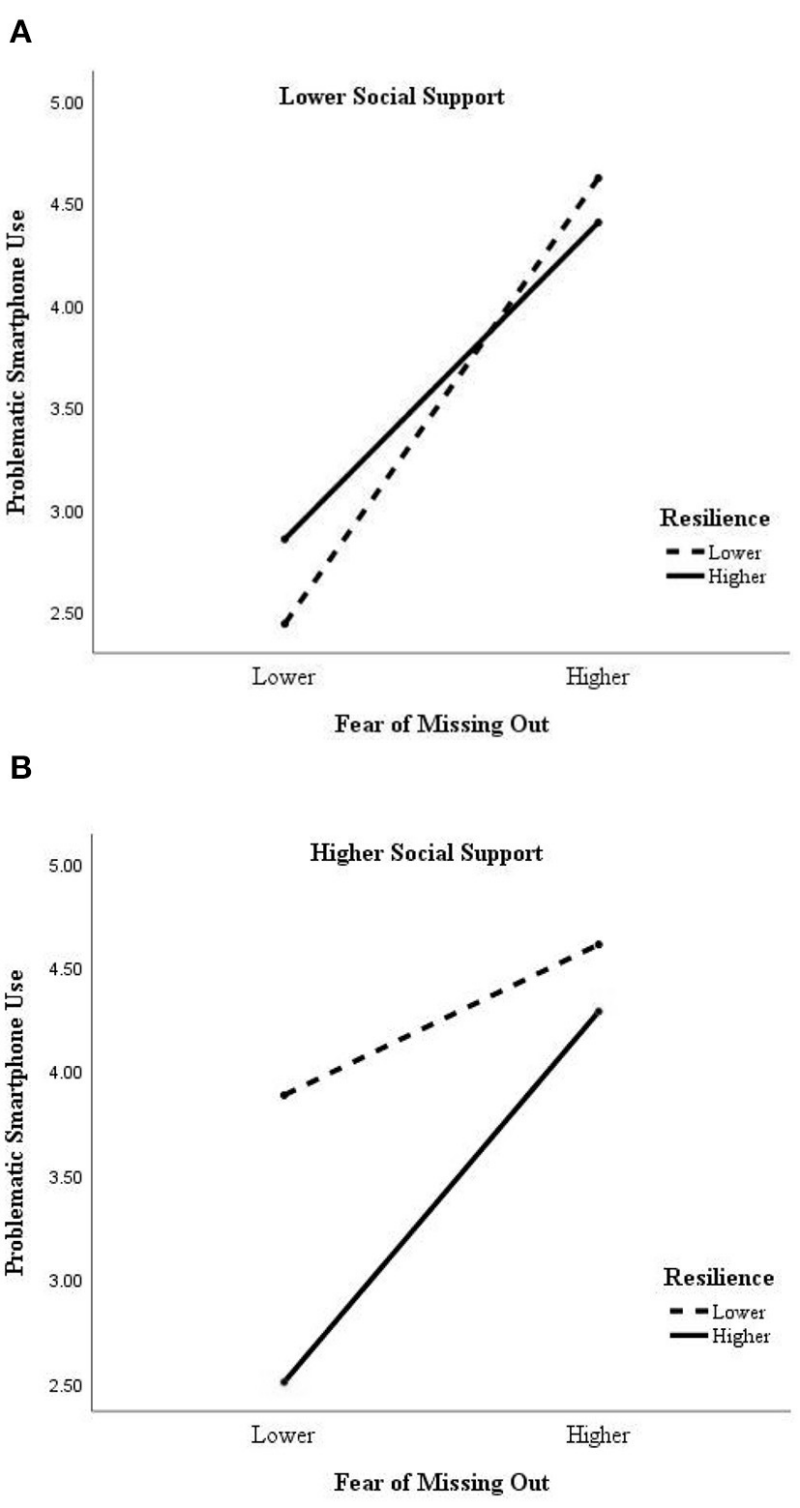
**(A)** The moderating effect of resilience between FoMO and problematic smartphone use under lower social support. **(B)** The moderating effect of resilience between FoMO and problematic smartphone use under higher social support.

To further show the moderated moderating role of resilience and social support between FoMO and problematic smartphone use, this study referring to Hayes (34) categorized resilience, social support, and FoMO as higher group (M+1 *SD*) and lower group (M-1 *SD*) and plotted the moderated moderating effect diagram (see Figures 2A,B). When college students were at higher levels of FoMO, they had higher levels of problematic smartphone use under conditions with lower levels of both resilience and social support (*M* = 4.61, *SD* = 0.82), and lower levels of problematic smartphone use under conditions with higher levels of both resilience and social support (*M* = 4.29, *SD* = 0.94). It suggests that resilience and social support can effectively attenuate the effects of fear of missing out on problematic smartphone use.

To investigate the mechanisms and boundaries of the effects of perceived COVID-19-related restrictions on mental health problem, this study used SPSS PROCESS 3.3 to construct a moderated mediation model (Model 18), and the results are shown in Table 4. Perceived COVID-19-related restrictions was positively associated with mental health problem among college students (β = 0.28, *p* < 0.001), therefore H1b is accepted. Perceived COVID-19-related restrictions was also positively associated with fear of missing out (β = 0.60, *p* < 0.001), and fear of missing out was positively associated with mental health problem (β = 0.36, *p* < 0.001), thus H2 and H3b are accepted. At the meantime, the direct effect of perceived COVID-19-related restrictions on mental health problem was not significant under the influence of FoMO (β = 0.02, *p* > 0.05). It indicates that fear of missing out fully mediates the association between perceived COVID-19-related restrictions and mental health problem among college students. Moreover, a 3-way interaction of FoMO, resilience and social support was positively associated with mental health problem (β = 0.14, *p* < 0.01), which means that the resilience and social support levels of college students moderate the relationship between fear of missing out and mental health problem, and H4b is accepted.

**Table 4:** The moderated moderated mediating tests of mental health problem.

**Outcome**	**Predictors**	**Fit Indices**		**Coefficients**	
		***R* ^2^**	***F***	**β**	***t***
Mental health problem	COVID-19-related restrictions	0.21	52.57^***^	0.28	9.82^***^
	Gender			−0.23	−7.70^***^
	Age			0.11	2.92^**^
	Grade			−0.13	−3.51^***^
	Duration of closure			−0.21	−6.96^***^
FoMO	COVID-19-related restrictions	0.38	121.25^***^	0.60	23.61^***^
	Gender			−0.08	−3.15^**^
	Age			0.06	1.83
	Grade			−0.11	−3.31^**^
	Duration of closure			−0.11	−4.11^***^
Mental health problem	COVID-19-related restrictions	0.50	79.84^***^	0.02	0.51
	FoMO			0.36	11.16^***^
	Resilience			−0.20	−5.62^***^
	Social support			−0.27	−7.99^**^
	FoMO × resilience			−0.05	−1.19
	FoMO × social support			0.02	0.52
	Resilience × social support			−0.02	−6.39^***^
	FoMO × resilience × social support			0.14	3.10^**^
	Gender			−0.14	−5.89^***^
	Age			0.05	1.54
	Grade			−0.06	−2.06^*^
	Duration of Closure			−0.14	−5.56^***^

* p <0.05,

** p <0.01,

*** p <0.001.

**Figure 3:**
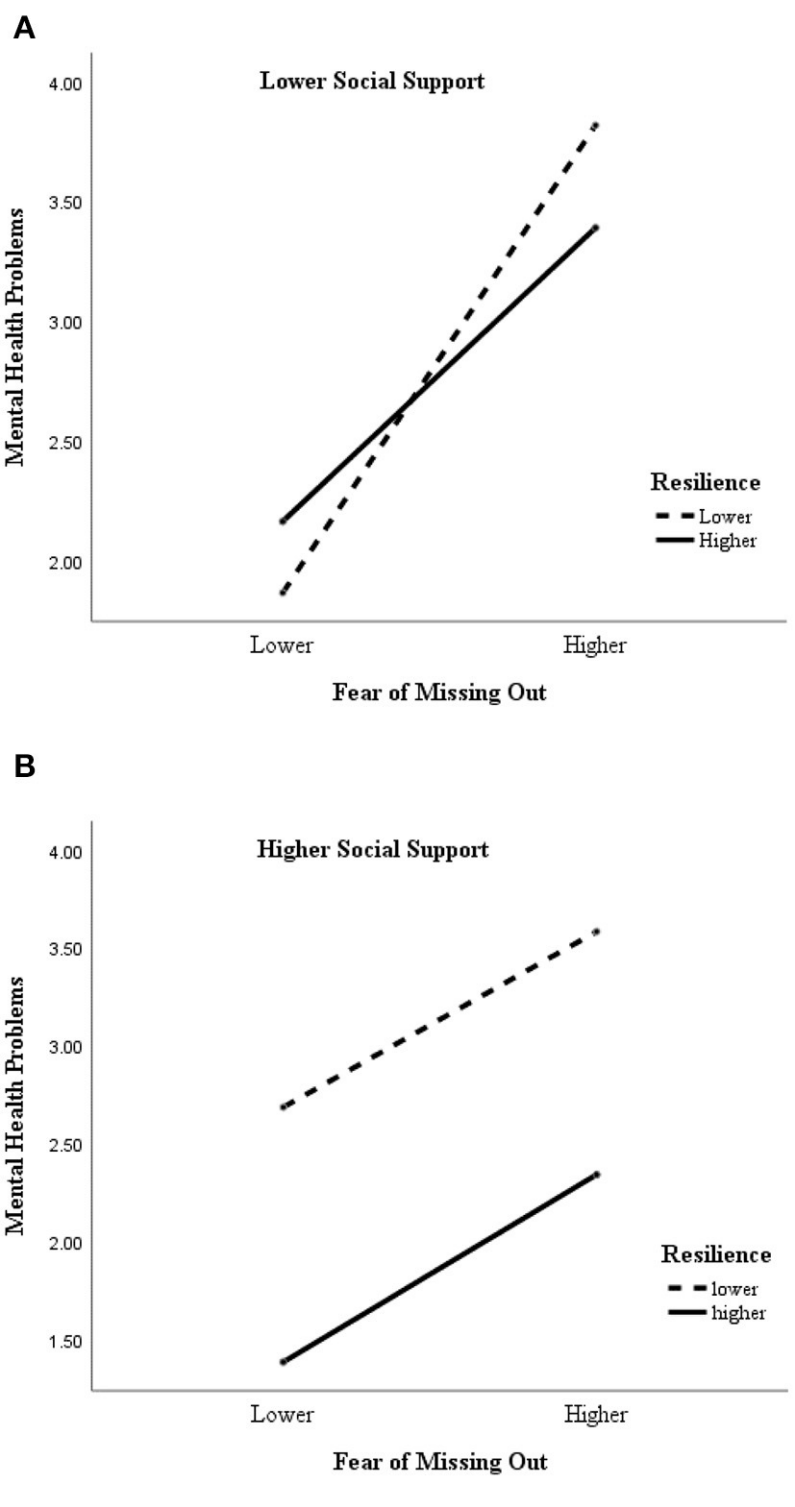
**(A)** The moderating effect of resilience between FoMO and mental health problem under lower social support. **(B)** The moderating effect of resilience between FoMO and mental health problem under higher social support.

To further show the moderated moderating role of resilience and social support between FoMO and mental health problem, this study referring to Hayes (34) categorized resilience, social support, and FoMO as higher group (M+1 *SD*) and lower group (M-1 *SD*) and plotted the moderated moderating effect diagram (see Figures 3A,B). When college students were at higher levels of FoMO, they had higher levels of mental health problem under conditions with lower levels of both resilience and social support (*M* = 3.82, *SD* = 0.42), and lower levels of mental health problem under conditions with higher levels of both resilience and social support (*M* = 2.34, *SD* = 1.12). It suggests that resilience and social support can effectively attenuate the effects of fear of missing out on mental health problem.

## Discussion

### Impact of COVID-19-related restrictions on problematic smartphone use and mental health among college students

In this study, we constructed two moderated moderated-mediation models based on self-determination theory and conservation of resources theory as a way to explore potential mechanisms between COVID-19-related restrictions and problematic smartphone use and college students' mental health levels. The results showed that perceived COVID-19-related restrictions is positively associated with problematic smartphone use and mental health problems among college students, thus supporting our hypothesis, which is consistent with previous findings. COVID-19-related restrictions may contribute to a lack of specific needs, triggering problematic smartphone use and consequently emotional stress changes (6). In our preliminary analysis, we found that men had higher levels of problematic smartphone use and mental health problems than women. This is similar to previous research findings that men are more likely to use social media than women when faced with FoMO (15). In terms of grade level, postgraduate students scored significantly lower than undergraduate students on problematic smartphone use and mental health problem levels. This may be because postgraduate students are more resilient and more self-determined than undergraduate students after the entrance exams and the postgraduate study life. For the duration of closure, the reason for the lower score on problematic smartphone use and mental health problems of “two months and over” is perhaps because of the students' adaptability to the environment and the fact that as time passes (35), the supporting measures of the school as well as the government will become more and more complete compared to the sudden situation at the beginning, which reduces the impact of the COVID-19-related restrictions on college students.

### The mediating effect of FoMO

In problematic smartphone use model, FoMO partially mediated the relationship between perceived COVID-19-related restrictions and problematic smartphone use. The results supported that perceived COVID-19-related restrictions is positively associated with FoMO, FoMO mediates the association between perceived COVID-19-related restrictions and problematic smartphone use among college students, and indicated that FoMO had an important influence on problematic smartphone use during the epidemic closure, which is consistent with previous research (17). According to self-determination theory, epidemic closure disrupts the rhythm of students' lives, and college students are fundamentally different from junior and senior high school students in that junior and senior high school students are subject to certain restrictions in their daily lives even if they do not undergo epidemic closure (36). Research has shown that individuals with high levels of FoMO desire to satisfy their basic psychological needs through excessive smartphone and social media use (37). The greater the perceived stress caused by the restrictions of the epidemic, the greater the level of basic needs missing, and the more individuals experience impaired self-regulation, i.e., FoMO. Thus, FoMO is a significant predictor of the association between COVID-19-related restrictions and problematic smartphone use.

In mental health problems model, FoMO fully mediated the relationship between perceived COVID-19-related restrictions and mental health problems. The results support our hypothesis that FoMO significantly mediates the relationship between COVID-19-related restrictions and mental health problems such as anxiety, depression and stress, meaning that for college students during the epidemic, the more stressful the perceived COVID-19-related restrictions are, the more likely they are to lead to FoMO, which indirectly leads to mental health problems. This is similar to previous research findings that FoMO, as a subtype of anxiety, can lead to mental health problems (38). According to Wortham, FoMO is a source of negative emotions such as depression, as individuals with FoMO are always anxious about missing out on novel experiences or other things. The COVID-19-related restrictions have deprived college students of many enjoyable experiences, such as outings, parties and friendships, making them more susceptible to this particular anxiety, which Chai et al. suggest makes them more sensitive to stressful events in their daily lives based on the social selectivity hypothesis (15). The results of the study are consistent with the finding that FoMO often fully mediates the relationship between variables related to psychological needs (39, 40).

### Moderated moderating effects of resilience and social support

The results show that the study supported that resilience and social support play a significant moderated moderating role between FoMO and both problematic smartphone use and mental health problems. See Figure 2, in problematic smartphone use model, resilience and social support had a significant interaction with FoMO. Students with high resilience had lower scores for problematic smartphone use when FoMO was high, and according to the results, scored lowest with higher levels of both resilience and social support. That is, these students used their resilience and social support as resources to alleviate the stress caused by FoMO and avoid problematic behavior. This result validates the scientific validity of resilience as a protective factor, and therefore, for future planning and setting up of mental health education on university campuses, enhancing students' resilience should be incorporated as an important component in the teaching design to improve resilience to frustration and environmental adaptability as a way to resist the subsequent changing epidemic environment.

See Figure 3, in mental health problems model, resilience and social support had a significant interaction with FoMO, which is consistent with previous research and our hypothesis. Even though the social support received during the epidemic closure was low, the previous personal upbringing had created and possessed a high level of resilience, i.e., protective resources. Therefore, when there is a change in the environment and higher anxiety is felt, it is still possible to maintain a better state of mind. And according to the results, scored lowest with higher levels of both resilience and social support. This accurately provides evidence to support the role of social support as a psychological protective factor during an epidemic (41). As an important formative factor of resilience, social support can work in tandem with resilience to reduce the impact of FoMO collectively and directly on mental health problems and problematic smartphone use. Social support can compensate for the lack of relational, autonomy and competence needs caused by FoMO, for example, by increasing the number of social and recreational activities such as recreation and sports in schools to enhance students' sense of competence and communication needs and by increasing students' freedom of choice in their closed lives to compensate for their autonomy needs.

### Limitations and future directions

The present study has some limitations. First, although we emphasized in the guideline that question responses are not right or wrong and are confidential, there may still be a social approval effect for the self-report scale, and follow-up studies could develop more objective measures of the relevant variables, including experimental methods. Second, this study is a cross-sectional study. In the future, a follow-up study could be used to examine the long-term benefits of resilience and social support on the mental health of college students during the epidemic. Finally, follow-up research could be conducted across cultures, and we can test whether the positive effects of resilience and social support on the mental health of college students in the context of the epidemic are consistent across cultures.

## Conclusion

This study investigates the effects of COVID-19-related restrictions on problematic smartphone use and mental health among college students. The perceived COVID-19-related restrictions may have influenced the development of problematic smartphone use and mental health problems among college students by triggering individual FoMO. FoMO is an important factor in triggering problematic smartphone use and mental health problems during the current epidemic, which provides an important entry point for the development of student mental health intervention strategies under the regular management of the epidemic in the future. This study explored how to alleviate the negative effects of individuals' FoMO triggered by the current COVID-19-related restrictions by constructing two moderated moderated-mediation models. This study found that resilience is a key individual resource for individuals to mitigate the negative effects of loss anxiety, while social support is an important environmental condition for individuals to mitigate the negative effects of loss anxiety. Therefore, nurturing students' resilience and creating an environment with a high level of social support are conducive to the psychological health development of college students under the regular management of the epidemic in the future.

## Data availability statement

The raw data supporting the conclusions of this article will be made available by the authors, without undue reservation.

## Ethics statement

The studies involving human participants were reviewed and approved by the Institutional Review Board, Normal College, Qingdao University. The patients/participants provided their written informed consent to participate in this study.

## Author contributions

ZG, YL, and XJ designed, performed, analyzed the research, and wrote the research. QQ critically reviewed and edited the manuscript. JL and YS made substantial revisions. All authors contributed to the article and approved the submitted version.

## Funding

This work was supported by the major projects of National Social Science Fund of China: “Research on the Construction of China's Marine Talent Ecosystem and the Construction of Dynamic Database” (No. 20&ZD130).

## Conflict of interest

The authors declare that the research was conducted in the absence of any commercial or financial relationships that could be construed as a potential conflict of interest.

## Publisher's note

All claims expressed in this article are solely those of the authors and do not necessarily represent those of their affiliated organizations, or those of the publisher, the editors and the reviewers. Any product that may be evaluated in this article, or claim that may be made by its manufacturer, is not guaranteed or endorsed by the publisher.
